# Surgical Outcomes and Associated Morbidity of Active and Expectant Management of Second-Trimester Placenta Accreta Spectrum (PAS)

**DOI:** 10.3390/medicina61010113

**Published:** 2025-01-14

**Authors:** Jessian L. Munoz, Rachel Counts, Amanda E. Lacue, Kayla E. Ireland, Patrick S. Ramsey, Kristyn Brandi

**Affiliations:** 1 Department of Obstetrics and Gynecology, Division of Maternal Fetal Medicine, Division of Fetal Intervention, Baylor College of Medicine and Texas Children’s Hospital, Houston, TX 77030, USA; 2 Department of Obstetrics & Gynecology, University of Texas Health Sciences Center at San Antonio, San Antonio, TX 78229, USA; countsr@uthscsa.edu (R.C.); ireland@uthscsa.edu (K.E.I.); ramseyp@uthscsa.edu (P.S.R.); 3 Department of Obstetrics, Gynecology and Reproductive Health, Rutgers New Jersey Medical School, Newark, NJ 07103, USA; mandy.lacue@gmail.com (A.E.L.);

**Keywords:** abnormal placentation, cesarean hysterectomy, cesarean section, maternal morbidity, placenta accreta

## Abstract

*Background and Objectives:* Management of second-trimester placenta accreta spectrum (PAS) is currently center-dependent with minimal evidence-based practices. This study aims to analyze outcomes of hysterectomy as second-trimester active management (AM) versus cesarean hysterectomy as expectant management (EM) in cases of PAS with intraoperative and postoperative outcomes. *Materials and Methods:* This study is a retrospective case-control study of patients with a pathology-confirmed diagnosis of PAS managed at a single center over 16 years (2005–2020). All cases were diagnosed during the first or second trimester by ultrasonography and managed by the same multidisciplinary team with delivery within the second trimester. *Results*: Thirty-four patients with PAS were diagnosed and delivered by the second trimester. Of these, (41.1%) elected for active management and 20 (58.9%) for expectant management but ultimately required delivery prior to 28 weeks’ gestation. Baseline demographics were similar between groups. Intraoperatively, no differences were noted in operative time (191.5 vs. 203 min, *p* = 0.85), blood loss (2300 vs. 2600 cc, *p* = 0.85), or incidental cystotomy (1 vs. 7, *p* = 0.10). Postoperative length of stay was similar (3 vs. 3.5 days, *p* = 0.28), and ICU admission was not statistically different (6 vs. 12, *p* = 0.48). *Conclusions*: This retrospective study suggests that when hysterectomy is planned, there is no difference in maternal outcomes and morbidity with an expectant management with cesarean hysterectomy in the second trimester compared to proactive cesarean hysterectomy.

## 1. Introduction

Placenta accreta spectrum (PAS) describes abnormal uterine attachment secondary to trophoblastic invasion within the myometrium [1]. Prevalence rates range from 0.01–1.1%, with the strongest association with patients who have a history of cesarean section and placenta previa. The risk of PAS in patients with a known previa increases with increasing number of cesarean deliveries [2]. Complications associated with placenta accreta are serious, including massive hemorrhage, peripartum hysterectomy, disseminated intravascular coagulopathy, and maternal death [3,4,5]. Risks for those undergoing active management or termination of pregnancy (TOP), particularly in the second trimester, are similar and include hemorrhage and possible hysterectomy [6,7].

A diagnosis is typically made from ultrasonographic findings, which is the primary imaging modality to characterize the degree of placental myometrial extension from accreta to percreta [8,9,10]. Diagnostic findings suggestive of PAS on ultrasound include the presence of placenta lacunae, interruption of the posterior bladder wall to uterine interface, lack of retroplacental clear space, increased vascularity in the bladder wall adjacent to the uterus, and myometrial thickness <1 mm. Secondary to the increased cost and provider expertise required for interpreting MRI findings of PAS, MRI is typically only utilized in cases where the diagnosis is questioned or requires further delineation [11,12]. Ultrasound imaging suggestive of PAS can be identified as early as 16–19 weeks’ gestation, with many patients identified at ultrasound performed at 18–20 weeks [13].

Management of those undergoing delivery with PAS can include cesarean delivery with conservative management, leaving the placenta in situ, or cesarean hysterectomy [14,15]. For patients undergoing second-trimester pregnancy termination, management options include gravid hysterectomy, hysterotomy, dilation and evacuation, uterine artery embolization, or medical management with adjuvant treatment [16,17]. Limited research has been performed in evaluating the difference in outcomes between those undergoing terminations versus expectant management for placenta accreta spectrum. Recent studies reported the success of UAE in second-trimester PAS termination, although vaginal delivery rates were 62% (31/51); therefore, the underlying pathology of this patient population may vary from traditional PAS pathology and management [18]. Thus, we aimed to describe the surgical and postoperative outcomes of patients with second-trimester PAS managed with active or expectant management.

## 2. Materials and Methods

### 2.1. Data Collection

We conducted a retrospective cohort analysis of women who presented to our center for the Placenta Accreta Spectrum program between January 2005 and December 2020, the time period of electronic medical record at our institution. Cases were identified by referral center database as well as by ICD-10 code O43.2 for placenta accreta. Institutional review board (IRB) approval was obtained prior to obtaining patient information from electronic medical records. Inclusion criteria included maternal age between 18 and 55 years with a viable pregnancy and antenatal suspicion for PAS by sonographic findings, MRI, or increased a priori risk based on maternal comorbidities such as presence of previa and multiple prior cesarean sections.

Final patient inclusion was dependent on pathology confirmation of PAS by a board-certified pathologist with extensive gynecologic experience. Exclusion criteria were the following: incomplete electronic medical records, delivery at an outside institution, and delivery without antenatal suspicion or imaging. Within the program, patients were managed by a multidisciplinary team as described above [19]. Active management was defined as medically indicated delivery or elective termination of pregnancy, and expectant management was defined as planned 34–36 w delivery after second-trimester diagnosis of PAS, but with delivery within the second trimester (unplanned).

### 2.2. Surgical Management

Current recommendations for PAS management include planned cesarean hysterectomy, ideally with an experienced team. Our center does not offer conservative management of PAS or uterine reconstructive procedures; thus, all cases were managed by cesarean hysterectomy.

Our Placenta Accreta Spectrum program provides multidisciplinary team management of PAS, including maternal–fetal medicine, gynecologic oncology, urology, and interventional radiology. As per our algorithm, the suspected cases of placenta percreta by ultrasonography or MRI are managed with cesarean delivery immediately followed by uterine artery embolization and hysterectomy. Patients with suspected placenta accreta (without serosal or parametrial involvement) undergo cesarean hysterectomy without uterine artery embolization [20].

### 2.3. Statistical Analysis

Normal distribution was determined by a Shapiro–Wilk test value greater than 0.05. Pearson’s chi-square test (χ^2^), Fisher’s exact test, Mann–Whitney U test, and T-test were applied when appropriate. Categorical factors were summarized using frequencies and percentages, while continuous measures were summarized using means ± SD or median and range as appropriate. *p*-values < 0.05 were considered significant for two-tailed analysis. Statistical analysis was performed using Graphpad software (version 9, Boston, MA, USA).

### 2.4. Data Management

Data were collected and stored using a REDCap electronic data capture tool hosted at our institution. REDCap (Research Electronic Data Capture, Vanderbilt University, Nashville, TN, USA) is a secure, web-based application designed to support data capture for research studies (https://redcap.vanderbilt.edu). STROBE guidelines were followed throughout this study [21].

## 3. Results

During the 16-year time period (2005–2020), 125 cases of pathology-confirmed PAS were managed by the multidisciplinary PAS team at our institution. Thirty-four patients (27.2%) were diagnosed and delivered within the second trimester. Of the patients diagnosed with placenta accreta spectrum (PAS) in the second trimester in this study, 14 (41.1%) elected for active management (AM) and 20 (58.9%) for expectant management (EM). There was a significant difference in gestational age at diagnosis, with the AM group having a lower average gestational age at diagnosis than the EM group (16.1 ± 4.9 vs. 19.7 ± 2.0 weeks, respectively, *p* = 0.007) (Table 1). In regard to background demographic data, however, there were no significant differences. Of the 34 patients included in this study, 86% of the AM group and 95% of patients from the EM group reported a history of CD, and the average number of CDs were similar between the two groups (2 vs. 2, *p* = 0.96). Further, 29% of the AM group and 25% of the EM group reported a history of D&C.

Our study aimed at understanding the intraoperative complications and postoperative outcomes between these groups. Gestational age at delivery and intraoperative characteristics were similar between the two groups. Mean operative time did not significantly differ between the AM vs. EM groups (191.5 vs. 203 min, respectively, *p* = 0.85). Similarly, the mean EBL was not statistically different (2300 cc vs. 2600 cc, *p* = 0.85) (Table 2). Intraoperative injury rates were also similar, with intentional cystotomy occurring in two patients (2 vs. 0, *p* = 0.16), incidental cystotomy in eight patients (1 vs. 7, *p* = 0.10), and ureteral injury in two patients (0 vs. 2, *p* = 0.5).

Postoperative data were similar between the two groups. The pathology results did not significantly differ between the AM vs. EM groups, with 7 found to have accreta (4 vs. 3, respectively, *p* = 0.41), 10 with increta (2 vs. 8, respectively, *p* = 0.14), and 17 with percreta (8 vs. 9, respectively, *p* = 0.72) (Table 2). The average length of postoperative stay was 3 days in the AM group and 3.5 days in the EM group (*p* = 0.28). Eighteen of the thirty-four patients required ICU admission postoperatively, but there was no statistical difference in ICU admission rates (6 vs. 12, *p* = 0.48) or average length of ICU stay (1 vs. 1 day, *p* = 0.68) (Table 3).

## 4. Discussion

Management of PAS is complex and is often associated with significant morbidity. Studies show that despite planned cesarean hysterectomy in the third trimester, half of all cases occur prior to this recommendation [22]. Thus, the challenge of second-trimester management presents a delicate balance between maternal health, neonatal outcomes, patient autonomy, and morbidity. Our study investigated surgical outcomes and morbidity in patients with PAS who underwent expectant management and termination of pregnancy. This study was conducted in a population with no significant differences in demographic factors, and similar outcomes amongst the two groups were shown. All patients were delivered at our institution, and operative characteristics were available for analysis. These operative characteristics included no significant difference in operative time, estimated blood loss, genitourinary injury, or degree of invasion of PAS. Similarly, there was no difference in postoperative length of stay or postoperative adverse outcomes. The only significant difference between the groups was earlier gestational age of diagnosis for patients undergoing active management.

There are little data elsewhere evaluating these outcomes, which are instrumental to patient care. Different management options have been suggested to help decrease EBL in patients with PAS undergoing second-trimester abortion, including the use of prophylactic uterine artery embolization. Further studies should be conducted to determine whether differing techniques for second-trimester PAS differ in EBL, a major morbidity associated with PAS. Further studies should also continue to look into if all procedures performed in patients with PAS have equal rates of injury. There is a well-known association of PAS and GU injury, with up to 29% of procedures described as resulting in urinary tract injuries [23,24]. These injuries, with previously identified risk factors found to be depth of invasion and number of prior cesarean deliveries, were found in our patient population across both groups.

Our study has several strengths and limitations that should be acknowledged. As a single-center study, uniform adherence to standard protocols at our center was possible with strict inclusion of complete medical records and gold-standard diagnosis by histopathology. By contrast, our center’s protocols and team availability may not reflect other centers, including our lack of reconstructive approaches, which may be routine at other centers.

From a research perspective, the prospective evaluation of second-trimester deliveries for PAS remains an area of uncertainty [7]. While many of these cases will be secondary to maternal–fetal instability, the factors to predict this, use of interventions, psychological impact, and maternal long term outcomes remain unknown.

There is a consensus in the literature highlighting the importance of a multidisciplinary team with specific resources, including blood banking, for the management and surgical planning of PAS. Multidisciplinary teams have been shown to reduce overall maternal morbidity including blood loss and ICU admission [15,24]. These outcomes remain significantly improved even in the setting of emergent deliveries [25]. In 2024, several institutions across the US recommended specific infrastructure for multidisciplinary teams [26]. As PAS continues to increase in the setting of increased cesarean deliveries, it is important for providers to have information regarding the risks of different outcomes. Multidisciplinary teams should include providers that can manage continuing pregnancies for those choosing expectant management and providers that can provide second-trimester abortion care and active management, if the patient decides for active management.

This study collectively shows cesarean hysterectomy performed for PAS, which is a procedure with associated surgical morbidity, and this morbidity is not confined to the trimester in which the procedure is performed. Thus, with appropriate team management, second-trimester delivery, whether planned or incidental, is associated with similar morbidity while being patient-centered and consistently working to improve outcomes.

## 5. Conclusions

This retrospective study suggests that when hysterectomy is planned, there is no difference in maternal outcomes and morbidity with an expectant management with planned cesarean hysterectomy at 34–36 weeks but a delivery in the second trimester compared to proactive management in the second trimester. Counseling options to provide patient-centered care for both termination and expectant management require a discussion of surgical risks, which may provide more information. It is of the utmost importance to be able to provide both patients and providers with critical information in the treatment of PAS disorders.

## Figures and Tables

**Table 1:** Demographics of the study population.

	Active Management (n = 14)	Expectant Management (n = 20)	*p*-Value
Age (years)	30.0 ± 6.0	31.6 ± 4.2	0.37
BMI (kg/m^2^) Gravidity Parity Prior CD History of D&C Number of prior CD Gestational age at diagnosis (weeks) Pregestational diabetes Chronic hypertension Anemia Public insurance	32.4 ± 5.4 4 [3, 7] 3 [2, 4] 12 (86) 4 (29) 2 [2, 3] 16. 1 ± 4.9 0 (0) 1 (7) 1 (7) 13 (93)	34.4 ± 6.9 4 [3, 5] 2 [2, 3] 19 (95) 5 (25) 2 [2, 3] 19.7 ± 2.0 3 (12) 3 (12) 5 (25) 15 (75)	0.37 0.69 0.77 0.55 1.00 0.96 **<0.01** 0.25 0.62 0.36 0.36

BMI = body mass index, CD = cesarean delivery, D&C = dilation and curettage. Values presented as mean ± SD, median [P25, P75], or N (column %); bold, *p* < 0.05.

**Table 2:** Operative characteristics.

	Active Management (n = 14)	Expectant Management (n = 20)	*p*-Value
Gestational age at delivery (weeks) Admission hemoglobin (g/dL)	21 [16, 25] 10.54 ± 1.7	24 [17, 26] 10.36 ± 1.3	0.61 0.74
Operative time (min) EBL (mL)	192 (149, 317) 2300 (1500, 6000)	203 (153, 401) 2600 (2000, 6750)	0.85 0.42
Component transfusion Whole blood Red blood cells Platelets Fresh frozen plasma Cryoprecipitate	2 (14) 12 (86) 4 (28) 6 (43) 1 (7)	3 (15) 17 (85) 7 (35) 12 (60) 3 (15)	1.0 1.0 1.0 0.48 0.62
Genitourinary injury Intentional cystotomy Incidental cystotomy Ureteral injury	2 (14) 1 (7) 0 (0)	0 (0) 7 (35) 2 (10)	0.16 0.10 0.50
PAS pathology Accreta Increta Percreta Postoperative LOS (days)	4 (28) 2 (14) 8 (57) 3 [3, 4]	3 (15) 8 (40) 9 (45) 4 [3, 6]	0.41 0.14 0.72 0.28

EBL = estimated blood loss, GU = genitourinary, PAS = placenta accreta spectrum, LOS = length of stay. Values presented as mean ± SD, median [P25, P75], or N (column %).

**Table 3:** Postoperative outcomes.

	Active Management (n = 14)	Expectant Management (n = 20)	*p*-Value
Transfusion >4 units	8 (57)	11 (55)	1.0
ICU admission	6 (43)	12 (60)	0.48
Intraoperative acidosis	12 (86)	13 (65)	0.25
LOS >4 days	5 (36)	10 (50)	0.49
ICU LOS (days)	1 [0, 2]	1 [0, 2]	0.68
Infection	1 (7)	2 (10)	1.0
Reoperation	2 (14)	1 (5)	0.55

ICU = intensive care unit, LOS = length of stay. Values presented as mean ± SD, median [P25, P75], or N (column %).

## Data Availability

The data presented in this study are available on request from the corresponding author due to patient privacy.
